# Enrichment of megabase-sized DNA molecules for single-molecule optical mapping and next-generation sequencing

**DOI:** 10.1038/s41598-017-18091-6

**Published:** 2017-12-20

**Authors:** Joanna M. Łopacińska-Jørgensen, Jonas N. Pedersen, Mads Bak, Mana M. Mehrjouy, Kristian T. Sørensen, Peter F. Østergaard, Brian Bilenberg, Anders Kristensen, Rafael J. Taboryski, Henrik Flyvbjerg, Rodolphe Marie, Niels Tommerup, Asli Silahtaroglu

**Affiliations:** 1 Department of Cellular and Molecular Medicine, Faculty of Health and Medical Sciences, University of Copenhagen, Nørre Alle 14, Copenhagen, 2200 Denmark; 2 Department of Micro- and Nanotechnology, Technical University of Denmark, Ørsteds Plads 345a, Kongens Lyngby, 2800 Denmark; 3 NIL Technology ApS, Diplomvej 381, Kongens Lyngby, 2800 Denmark

## Abstract

Next-generation sequencing (NGS) has caused a revolution, yet left a gap: long-range genetic information from native, non-amplified DNA fragments is unavailable. It might be obtained by optical mapping of megabase-sized DNA molecules. Frequently only a specific genomic region is of interest, so here we introduce a method for selection and enrichment of megabase-sized DNA molecules intended for single-molecule optical mapping: DNA from a human cell line is digested by the NotI rare-cutting enzyme and size-selected by pulsed-field gel electrophoresis. For demonstration, more than 600 sub-megabase- to megabase-sized DNA molecules were recovered from the gel and analysed by denaturation-renaturation optical mapping. Size-selected molecules from the same gel were sequenced by NGS. The optically mapped molecules and the NGS reads showed enrichment from regions defined by NotI restriction sites. We demonstrate that the unannotated genome can be characterized in a locus-specific manner via molecules partially overlapping with the annotated genome. The method is a promising tool for investigation of structural variants in enriched human genomic regions for both research and diagnostic purposes. Our enrichment method could potentially work with other genomes or target specified regions by applying other genomic editing tools, such as the CRISPR/Cas9 system.

## Introduction

Structural variants (SVs) are genomic rearrangements that may lead to phenotypic diversity and human diseases^1^. No single technology can adequately capture all forms of structural variations to provide accurate and contiguous genome assembly^2,3^. Despite the enormous progress in NGS techniques, SVs remain difficult to detect reliably due to the short read lengths^3^. In addition, NGS sequencing platforms rely on the alignment of millions of short sequence reads^4^, thus NGS results require careful examination, particularly when investigating long-range genetic information^5^. Single-molecule DNA optical mapping, pioneered in mid-1990s by the Schwartz laboratory^6,7^, was first proposed as a powerful tool for genome sequence assembly in 1999^8^. Since then optical mapping has developed rapidly. It is nowadays used to improve the assembly of fragmented genome sequences that cannot be resolved by NGS methods^9^, as optical maps provide long-range scaffolds onto which short NGS reads can be assembled^10^. Thus, the long-range genome sequence information is preserved, enabling direct investigation of structural variants in sub- to 2 megabasepair-long single DNA molecules with a resolution of 1–5 kilobasepairs^11,12^. One important parameter in optical mapping is the degree of stretching of the DNA molecule. While high throughput methods exist, they either suffer from uneven or incomplete stretching (<85% of the contour length of the DNA molecule), compromising the optical mapping sensitivity. Stretching above 90% provides a sufficient resolution for single molecule sensitivity of the optical mapping. Such stretching can be achieved by manipulation of individual molecules^11,13–16^ but this lowers throughput. Methods that can enrich the regions of interest might circumvent the need for high throughput.

Methods of target enrichment, also termed reduced representation, are commonly used in NGS workflows to enrich for subsets of the genome before sequencing^17^. Target enrichment is a more cost-effective method compared to whole genome sequencing, as many genes can be sequenced simultaneously with higher coverage and deeper sequencing across many samples^18–20^. Customized sequencing of targeted genes opens new perspectives for implementing NGS as a routine tool in medical diagnostics laboratories, as it requires significantly less input material and offers straightforward data analysis^21^.

None of the existing target enrichment methods, commonly used in NGS workflows^17,22,23^ could be combined with subsequent optical mapping, since fragmentation of the target sequence to less than 5 kilobasepairs (kb) pieces is a key step for preparing DNA for any NGS analysis^24^.

**Figure 1:**
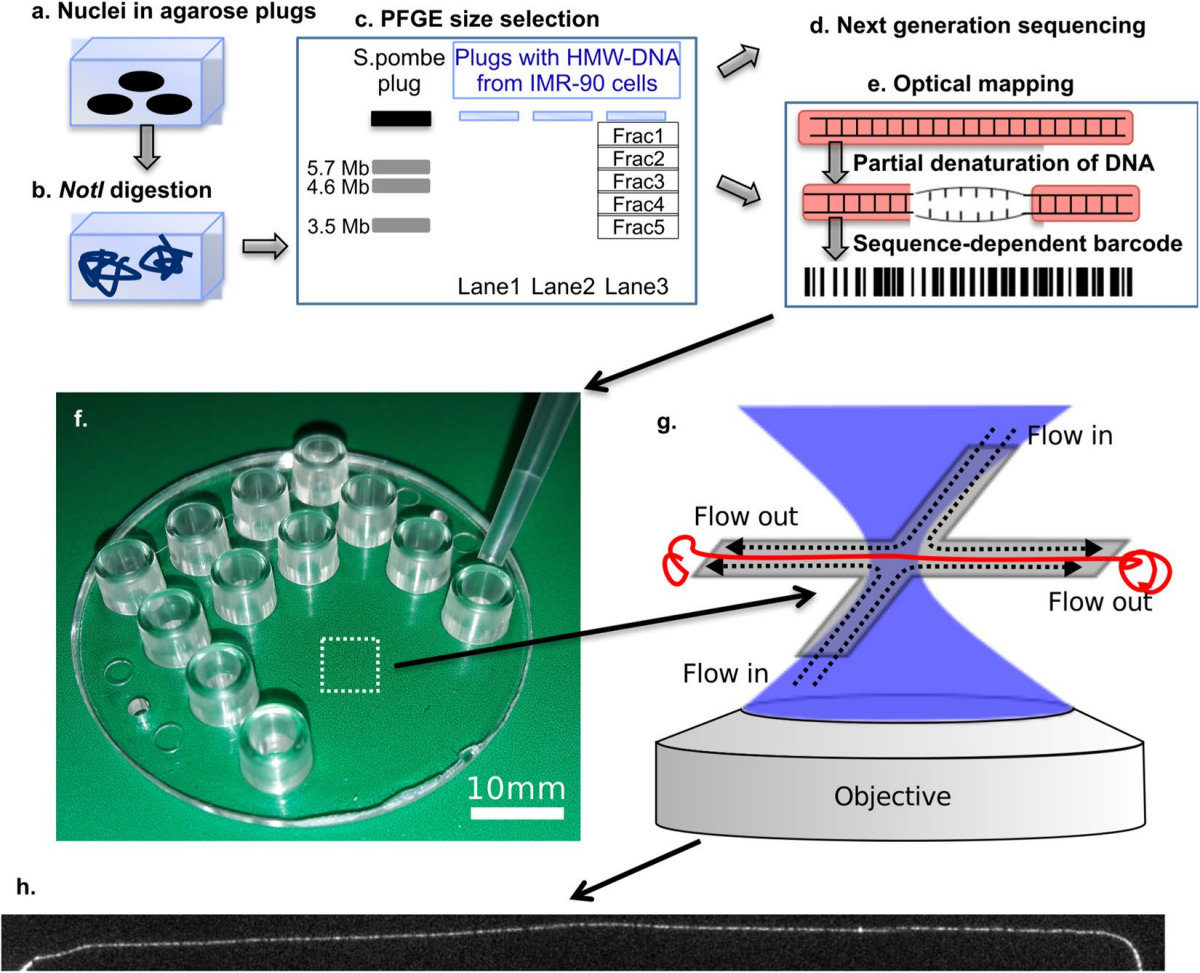
Schematic representation of the workflow for the enrichment method. **(a**) High molecular weight (HMW) DNA is isolated from IMR-90 cells within agarose plugs and (**b**) digested by NotI enzyme. (**c**) DNA fragments are size-separated by pulsed-field gel electrophoresis. (**d**) DNA fragments are sequenced with a short-read NGS method. (**e**) Some of these DNA molecules from the same gel are stained, partially denatured and renatured to create a fluorescent pattern (DR profile), stretched and visualized in the nanoslit device. (**f**) The chip is loaded with DNA molecules with DR patterns. (**g**) A megabasepair-long DNA fragment is flow stretched, imaged (**h**), and its DR map is compared with the theoretical melting profile of the reference genome (hg19). The image of the molecule covers approximately 0.7 Mb.

We hereby enrich for megabase-sized single DNA molecules from specified human genomic regions for optical mapping in nanodevices and NGS. Genomic DNA molecules longer than 0.5 megabase (Mb) are fragile and shear easily when stirred or pipetted^25^. In order to isolate intact, very long chromosomal DNA molecules, cell nuclei are embedded in agarose plugs, and digested with the rarely cutting restriction endonuclease NotI, followed by size selection of the DNA fragments by pulsed-field gel electrophoresis (PFGE), which enables separation of large DNA molecules up to 10 Mb in size^26^. Molecules, recovered from the PFGE gel, were analysed by NGS. Size selected DNA from the same PFGE gels were analysed by single-molecule optical mapping to demonstrate that the method indeed produced long, intact DNA molecules from regions specified by NotI-cutting. Figure 1 shows a schematic workflow.

Here we applied a DNA optical mapping technique called denaturation-renaturation (DR) mapping (Supplementary Information) that has some unique advantages compared to other optical mapping techniques, as *(i)* there is no need for site-specific labelling, and *(ii)* multifold coverage of the genome and construction of consensus maps are not required because the probability of aligning a DR map from a single 100 kbp molecule to an incorrect position in the human reference genome is negligible^11^. Single DNA molecules with DR patterns of bright (GC-rich) and dark (AT-rich) regions are loaded into the nanofluidic chip, stretched up to 98% of their contour length by a cross-flow, and imaged (Fig. 1, see also Supplementary Fig. S1). The black and white DR pattern on the DNA (Fig. 1h) resembles a barcode, which is used to locate the molecule within a reference genome^11,27,28^. The flow-stretch device used here to stretch DNA and image DR patterns has much lower throughput than commercially available optical mapping systems (e.g,, BioNano Genomics), which linearize nicked and fluorescently labelled genomic DNA molecules in nanochannel arrays^29,30^. Our enrichment method increases the efficiency of DR mapping and thus lowers the need for high throughput.

## Results

### Digestion with NotI enzyme

**Figure 2:**
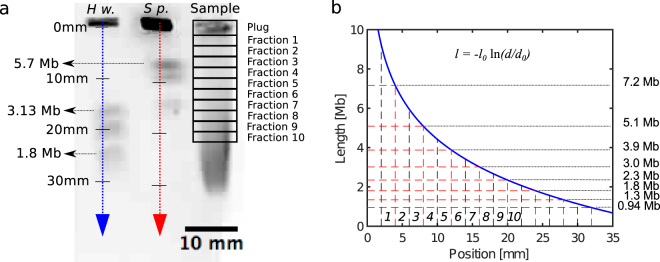
NotI-cut DNA extracted from gel. (**a**) PFGE lanes were sliced into 2 mm or 4 mm wide agarose pieces for NGS or single-molecule denaturation mapping, respectively (fraction 1–10). As size markers, CHEF DNA H. wingei (MHw, range 1.05–3.13 Mb) and CHEF S. pombe (MSp, range 3.5–5.7 Mb) were used (from Bio-Rad). The original unprocessed gel image is provided as Supplementary Figure S7 with the relevant corresponding bands denoted. (**b**) The length of the DNA molecules plotted versus the distance travelled in the gel. The vertical dashed black lines mark the cuts defining the fractions 1–10, and the horizontal lines define the ranges for the NotI groups.

The restriction enzyme NotI from *Nocardia otitidis-caviarum* with recognition sequence 5′-GC^GGCCGC-3′ has been reported to cut only upon recognition of two unmethylated CpG sites within the sequence (5′-GC^GGCCGC-3′)^31^. From the human reference genome and the methylation map, we define a NotI fragment as a genomic region between two NotI recognition sites with two unmethylated CpG sites. IMR-90 female human fetal lung fibroblasts was used in this study, since the single-base methylation data is available for this cell line in the UCSC Genome Browser. Thus the theoretical prediction of NotI cutting was performed taking the impact of methylation into consideration^32^. Theoretical NotI fragments longer than 1 Mb is 13.5% of the total number of fragments (Supplementary Fig. S2), but they cover almost 70% of the genome. The NotI fragments were divided into groups according to size. The groups correspond to the theoretical lengths from equidistant cuts of the gel with a separation of 4 mm (see Fig. 2b). Before applying the enrichment method presented in this paper, it is important to check the methylation status of the cytosines at the recognition sites of NotI (or of other used restriction enzymes) in the relevant cell line. Another challenge for enrichment protocols based on restriction enzymes is that they rely on cutting the genome at known positions, so cuts in regions with unknown sequence (e.g. telomeric regions) are not properly accounted for in the theoretical restriction maps.

### Size-selection with PFGE

Genomic DNA extracted from IMR-90 cells was digested with NotI within agarose gel plugs and separated according to size by pulsed-field gel electrophoresis (Fig. 2a and Supplementary Information). PFGE lanes were sliced into 2 mm or 4 mm wide agarose fractions for NGS or single-molecule denaturation mapping, respectively. For NGS analysis, 11 fractions were examined: the plug, and fractions 1–10 (Fig. 2). DNA from fractions 4–9 provided the most suitable molecule length for optical mapping in our nanofluidic chip design (see below and Supplementary Information). The distance *d* travelled by a DNA molecule in the gel is well-approximated by an exponential length dependence *d* = *d*_0_ *exp(−l/l*_0_), where *l* is the DNA length, *l*_0_ = 3 Mb is a characteristic length scale, and the prefactor is *d*_0_ = 44 mm. Inverting this expression, we find that DNA molecules that have travelled a distance *d* have a length *l* = *−l*_0_ *ln(d/d*_0_) for *d* < 44 mm. A plot of this expression is shown in Fig. 2b. We also observe that the spread in distances travelled by molecules of the same length is independent of the specific length (i.e., the widths of the Gaussians are independent of length). Thus we speculate that the spread is due to the size of the plug, and that using smaller plugs might lower it, and hence increase the precision of the method. From these calculations, we can estimate the size interval of the DNA molecules in each of the ten 2 mm wide fractions cut from the gel for NGS analysis, and the 4 mm wide fractions for DR mapping. In Fig. 2b, the alternating black and red, dashed vertical lines mark the separation of the ten different fractions cut from the gel. Red, dashed horizontal lines mark the separation used for the NotI groups.

### Single-molecule DR mapping of molecules from the gel

According to the analysis of the PFGE gel, molecules from fractions 4 and 5 should be in the intervals 4.4–5.1 Mb and 3.9–4.4 Mb, respectively (see Fig. 2b). We selected these two fractions as the main source of molecules for DR mapping to verify our selection and enrichment method. We used wider fractions (4 mm wide slices) compared to the NGS experiments (2 mm wide slices), as the DR mapping experiments depend crucially on long molecules, because molecules shorter than 0.7 Mb cannot be stretched in the device. We also extracted molecules from the double-fractions 6–7 and 8–9 in order to confirm that it is possible to obtain long molecules from other parts of the gel. We observed that the longest expected molecules had reduced lengths. The most likely reason is that these molecules break during handling after the PFGE separation, since it was reported that chopping gel-embedded megabased-sized molecules does not lead to any substantial damage^33^, as molecules do not remain fully stretched after the electrical field is switched off^34^. In total, 606 DNA molecules were extracted from the PFGE samples, captured, and imaged in the nanofluidic chip. All of the DR maps were then compared with an *in silico* theoretical DR map extrapolated from the sequence of the hg19 human reference genome, and 566 DR maps could be aligned unambiguously to specific positions in the human genome. The numbers of molecules both analysed and aligned are an order of magnitude higher than in previous studies (21 molecules in^11^, and 24 molecules in^28^). Moreover, 190 genomic regions were covered by more than one DR map due to the enrichment protocol described in this study (Supplementary Table S1). Finally, 40 of the 606 imaged molecules could not be mapped to any position in the human reference genome. Some of these showed clearly visible repeating blocks or regions. So the most likely reason is that these molecules originate from larger unannotated parts of the human genome, e.g. from centromeres or short arms of the acrocentric chromosomes^11,28^, that they contain repeated sequences not included in the reference genome, or simply contain errors, e.g., due to imperfect melting of the DNA.

### NGS analysis of DNA molecules from the PFGE gel

**Figure 3:**
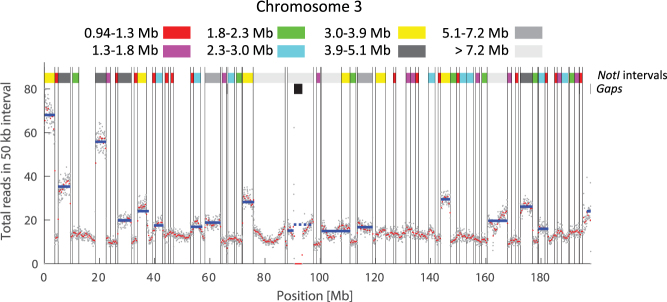
Histogram of NGS sequence reads mapped to chromosome 3. Grey dots mark the total number of reads in each 50 kb interval for DNA recovered from fraction 5 and aligned to chromosome 3. Red dots show the bin average for 400 kb bins. Full, blue horizontal lines marks regions defined as enriched. The coloured top band indicates the size and positions of the NotI fragments, and the colour shows which NotI group it belongs to. In the band below, the black region marks the positions and extents of the known gaps, including the metacentric gap. Enriched regions overlapping with gaps are excluded from the analysis and are marked with dashed, blue horizontal lines (e.g., around 90 Mb).

**Figure 4:**
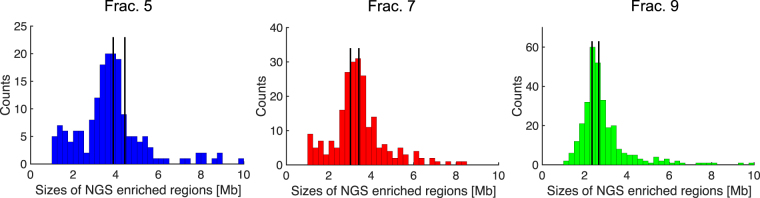
Histograms of the NGS enriched region sizes for the gel fractions 5, 7, and 9. The total number of counts is 178, 228, and 306, respectively. The full vertical lines show the expected length of the NGS enriched regions based on the DNA ladders in the gel (Fig. 2d).

DNA from each of the ten 2 mm wide fractions cut from the gel were extracted and sequenced on an Ion Proton sequencer. The average read length for the different fractions was 153 bp (see Supplementary Table S2), and the total number of reads matching the reference genome in each 50 kb interval was recorded (see Methods). As an example, the grey dots in Fig. 3 show the total number of reads in each 50 kb interval for DNA recovered from fraction 5 and with reads aligned to chromosome 3. Red dots show the bin average over a 400 kb interval. The thin, vertical black lines mark the separation of NotI fragments longer than 0.94 Mb, and the colors in the band at the top indicate their sizes. The base line for the number of counts in each interval is of the order of 15, but sharp plateaus with higher counts are clearly visible. These are our candidates for enriched regions (NGS enriched regions). It is striking how well the plateaus match the borders of the NotI fragments. We use a modified version of the CUSUM step-finder algorithm^35^ with a local mean and standard deviation^36^ to detect the plateaus. Regions with values above a threshold are counted as enriched, and their positions and extents are shown with horizontal, full blue lines in Fig. 3. The black bars in the row below the colored band denote the extent of the known gap in chromosome 3 in the hg19 human reference genome. Enriched regions overlapping with gaps are discarded from the analysis (horizontal, dashed blue lines). Size distributions of the NGS enriched regions for the fractions 5, 7 and 9 are shown in Fig. 4. All fractions have their main peak, representing the highest number of NGS enriched regions, within or near the theoretical range (full, red vertical line) based on the analysis of the gel (Fig. 2b). The theoretical range for fraction 5 is 3.9–5.1 Mb, fraction 7 – 3.0–3.9 Mb, and fraction 9 – 2.3–3.0 Mb. The main peaks have lower values for the fraction 9 than for the fractions 7 and 5, which are in good agreement with the expected PFGE separation – long molecules travel slower in the gel than shorter ones. The peaks in the size distributions are, however, broader than expected. Possible explanations are that the gel tends to collapse when cut. The motion of the DNA in the gel also depends on the specific lane, which might increase the widths of the size distributions of the NGS enriched regions. Moreover, grouping of the NGS reads in 50 kb intervals leads to overestimated island sizes: if two NotI fragments are separated by less than 50 kb, they will be counted as a single, larger fragment unless the number of reads on the two NGS enriched regions are so different that it is detected by the step-finder algorithm. Finally, the step-finder algorithm itself and the choice of the threshold can also influence the size distribution of the NGS enriched regions. We conclude that the two-step method for gel preparation indeed allows for size selection of DNA molecules by PFGE.

### Comparing positions of NGS enriched regions with NotI predicted fragments

**Figure 5:**
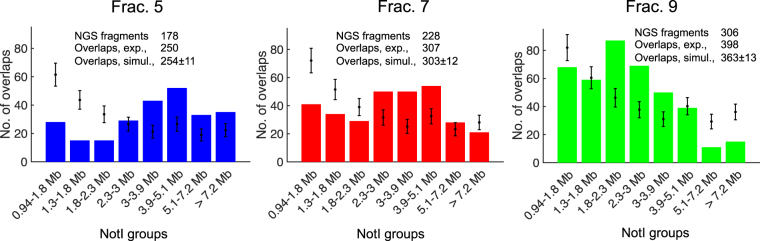
Histograms of NGS enriched regions with an overlap greater than 0.5 Mb with a NotI fragment in the specified range. Results are shown for fractions 5, 7 and 9. The total number of NGS enriched regions was 178, 228, and 306, respectively. Points with error bars show the average number of counts and the standard deviations from a random permutation of NGS enriched regions for the same number of NGS enriched regions and with the same size distribution as in the experiment. The total number of overlaps in the experiments and the simulations are stated in the figures.

When the enrichment method works, the NGS reads obtained from a given gel fraction should be enriched for the predicted NotI-fragments (Fig. 3). In this study, we define an NGS enriched region to overlap with a NotI fragment if the overlap is greater than 0.5 Mb. Then we recorded which NotI group, defined in Fig. 2b, the NotI fragment belonged to. Note that an NGS enriched region can overlap with more than a single NotI fragment, and that we only include NotI fragments longer than 0.94 Mb in the analysis. The number of NGS enriched regions overlapping with each of the NotI groups is shown in Fig. 5. Points with error bars show the average number of counts for each NotI group and its standard deviation from a random permutation of NGS enriched regions for the same number of NGS enriched regions and with the same size distribution as in the experiment (see Fig. 4). The total number of overlaps is almost identical for the experimental data and the simulated ones. For example, for fraction 5 the 178 experimental NGS enriched regions overlap with 250 NotI fragments, while a random permutation of 178 NGS enriched regions with the same size distribution gives on average 254 ± 11 overlaps with NotI fragments. The distribution of overlaps with the NotI groups is, however, different for the experimental and simulated data. For example, molecules from the gel fraction 5 overlap significantly more with NotI fragments in the size range 3.9–5.1 Mb than expected if the NGS enriched regions from this fraction were placed randomly on the genome. For molecules from fraction 9, the same holds for NotI fragments in the range 1.8–2.3 Mb. Thus, we conclude that there is a significant overlap between enriched regions in the NGS data and the predicted NotI groups. DNA is, however, fragmented before NGS sequencing, so we have not yet demonstrated if the DNA cut and extracted from the gel was totally intact. Our ability to perform DR mapping is an implicit confirmation that the molecules are still megabase-long after extraction from the gel since the flow-stretching procedure used for DR mapping requires such long molecules. We also observed a few short NGS enriched regions that overlap with long NotI regions (>5.1 Mb), e.g. in fraction 9 where overlaps with shorter NotI fragments are expected. This can occur by chance, but it might well be due to the breakage of the long molecules during the handling, migration in the gel^37^ or due to the enzyme’s so-called star activity. Under non-optimal conditions restriction endonucleases may digest sequences, which are different from their defined recognition sites.

### Comparing positions of aligned DR maps with predicted NotI fragments

**Figure 6:**
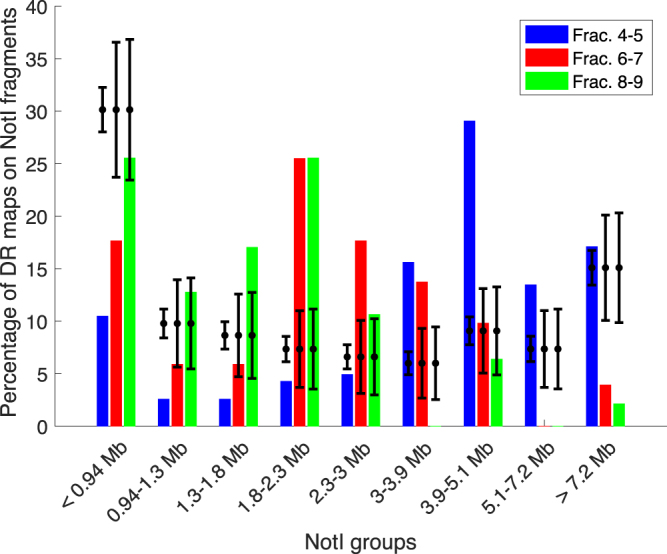
Overlaps between DR maps and the NotI groups for the three different fractions cut from the gel. The total number of counts is 468, 51, and 47 for fractions 4–5, 6–7 and 8–9, respectively. Points with error bars are the expected percentages of DR maps covering NotI fragments in each NotI group and their standard deviations estimated from binomial statistics.

Of the 566 DR maps from individual molecules that were successfully aligned to the hg19 human genome assembly, 468 molecules were extracted from the double-fractions 4–5, 51 molecules were from fractions 6–7, and 47 molecules were from fractions 8–9. The locations of these molecules were then compared with the positions of the different groups of NotI fragments. As DR maps are on average only 0.7 Mb long, a DR map is defined as overlapping with a NotI fragment if the center of the DR map is on the NotI fragment. So here a DR map can only overlap with a single NotI fragment. Results are illustrated in Fig. 6. Points with error bars show the mean and standard deviation of the expected percentage of overlaps. These are calculated from the fraction of the genome covered by NotI fragments in the given size interval and binomial statistics. As for the NGS enriched regions, DR maps recovered from the combined fractions 4 and 5 overlap with NotI fragments within the size range 3.9–5.1 Mb more frequent than expected by chance. For DR maps from gel fractions 6–7 and 8–9, we see a tendency that DR maps mainly overlap with slightly shorter NotI fragments than expected. This might be due to inaccurate slicing or a collapse of the gel. A χ^2^-test, which compares the observed number of overlaps for each NotI fragments groups with the expected number of counts, shows that it is very improbable that the results could happen by chance (one-sided *p*-values less than 10^−5^ for all three gel fractions). We conclude that the enrichment procedure gives an enhanced fraction of DR maps of NotI fragments of the specified sizes, and that the enrichment method indeed produces megabase-long DNA molecules after recovery from the gel.

### Examples of DR maps from unsequenced parts of the human genome

**Figure 7:**
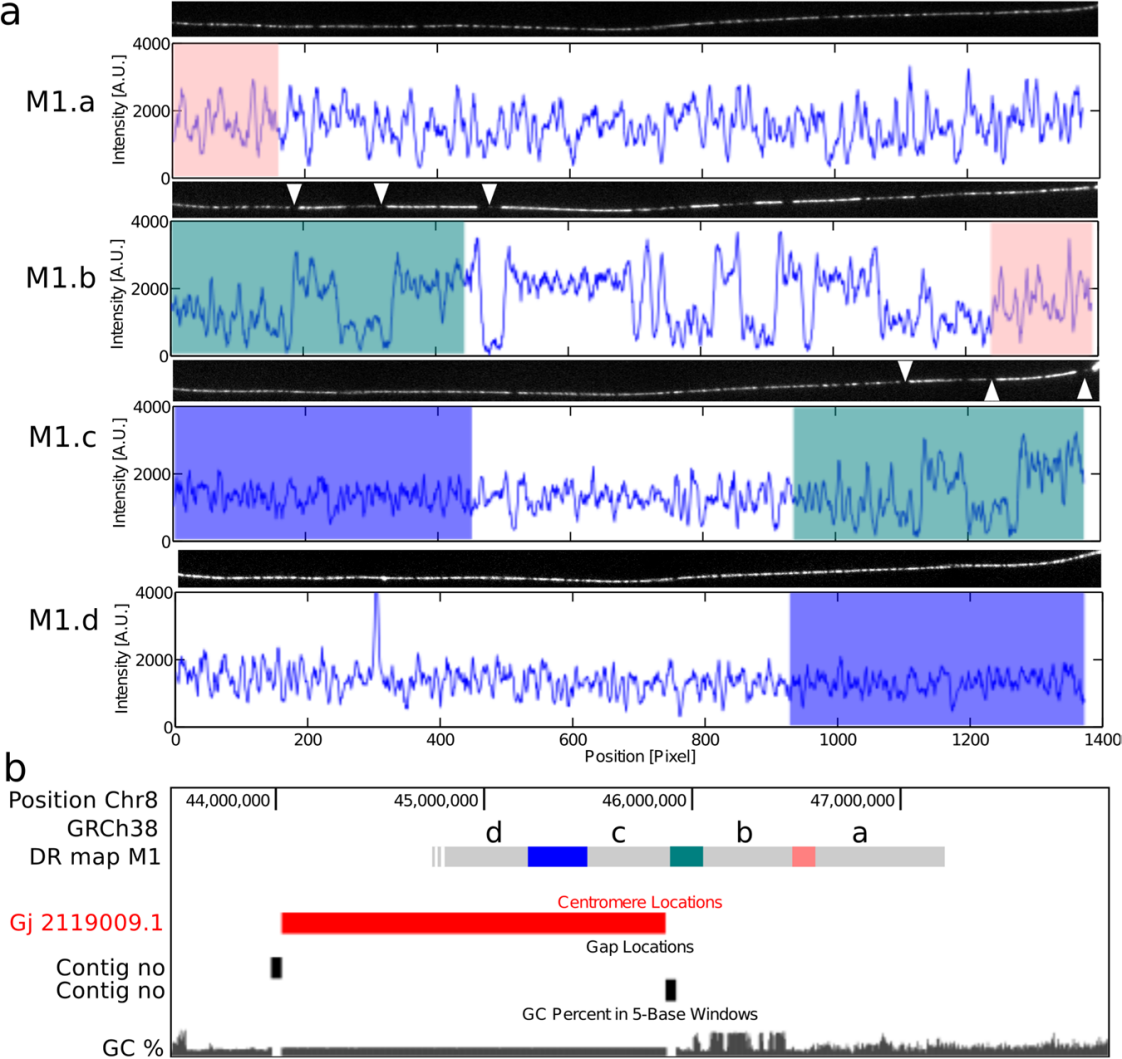
Optical map covering a gap on chromosome 8. (**a**) Optical DR maps and the corresponding intensity profile of the DNA for the four sub-maps M1.a-d. The colored regions overlap with regions of the same color. (**b**) Positions in chromosome 8 of the DR map M1.a–d, the centromere, and the GC-content in the hg38 reference genome.

As an application of the enrichment method, we show that DR maps can give coarse-grained but valuable information about poorly annotated regions of the genome. It is estimated that 5–10% of the human genome remain poorly characterized^38^. This figure agrees well with our detection of 40 out of 606 (6.6%) unalignable molecules. Especially interesting molecules would be those that border an annotated with an unannotated region, since it would provide a specific localization to the unannotated part. Indeed, we encountered one molecule, M1, which overlapped partly with one of the few annotated centromeric regions, on chromosome 8 (Fig. 7).

Molecule M1 was much longer than the length of the nanoslit, so it was visualized by sliding the molecule through the nanoslit in four steps, generating four individual DR maps M1.a-d (Fig. 7a). Features common to overlapping sub-maps were used to stitch them together to reconstitute a continuous map of M1. Some of these features are even visible with the naked eye, e.g., those marked with white triangles in Fig. 7a. Red, green, and blue rectangles indicate the overlaps between M1.a and M1b, M1.b and M1.c, and M1.c and M1.d, respectively. The alignment to the hg19 reference genome showed that parts of the molecule matched chromosome 8 near its centromeric region, which is defined as a gap. This gap has been closed in the more recent version of the human reference genome (hg38)^38^, so we made a query for alignment in the theoretical melting profile for this reference genome. Two of the M1-sub-maps still mapped uniquely to chromosome 8 (see locations in Fig. 7b). The sub-maps M1.c and M1.d could not be located, but sub-map M1.c overlaps with fragment M1.b, which is flanked by a centromeric region (Fig. 7a). This confirms that M1.d is located in the centromere, and thus that the DR maps M1.c-d provide a coarse-grained image of the melting profile of part of the chromosome 8 centromere. Remarkably, the DR map of the centromere show no repetitive features at the optical resolution (see Fig. 7a), as it could be expected considering the well-known repetitive nature of the centromere. Melting properties of DNA is related to the local GC content, which is shown as a black coverage plot in Fig. 7b. Outside the centromeric region, the GC content varies significantly, but it is constant in the centromere according to the hg38 human genome reference. In contrast, the melting profile of the DNA here shows significant variations (Fig. 7a, M1.c and M1.d), although the pattern is different from outside the centromere, i.e. it oscillates faster. Even more striking is the difference in melting temperature between the centromeric region and the flanking regions. With the software package Bubblyhelix (www.bubblyhelix.org)^39^ we calculated the fraction of closed basepairs (helicity) versus temperature inside the centromeric region and in two neighbouring regions of the same size (see Supplementary Fig. S3). Theoretically, the centromeric region clearly melts at a much lower temperature than its neighbouring regions, but in the experiment the intensity of the light emitted from the three regions are not significantly different (Fig. 7a). We conclude that the melting profile of the reference genome differs significantly from that of molecule M1. Most likely this is due to a notable difference in GC content. Another application of DR maps is for detection of repeated elements on the kb-scale (Supplementary Information).

## Discussion

Despite the enormous development of next-generation sequencing platforms, long-range genetic information from native, non-amplified DNA fragments is still lacking. Optical mapping operating on megabase-size DNA molecules can provide new insights into the genomic structure by providing a long-range scaffold for NGS short reads. However, all optical mapping methods rely on construction of ordered genomic maps from randomly selected molecules. Customized target enrichment methods that reduce genome representation provide many benefits, such as higher coverage of specified regions, more straightforward data analysis and lower running costs. Thus, target enrichment methods are of interest for both research and medical diagnostics purposes. Here we introduced a method for selection and enrichment of megabase-sized DNA molecules for single-molecule denaturation mapping and next-generation sequencing. Key components of the enrichment method are (i) preparation and handling of megabase-long DNA molecules, (ii) size-selection of megabase DNA molecules from NotI digested human genomes by PFGE, and (iii) subsequent recovery of the molecules from the gel without breakage. This enrichment method is the first attempt to analyse non-randomly selected single molecules from human cells by denaturation mapping in nanodevices. We did a comparison of the theoretical predictions for NotI cut DNA from the PFGE experiment, the NGS sequencing results, and the positions of the localized DR maps to validate the enrichment procedure. The analysis showed that we can indeed enrich for megabase-long molecules from specified regions in the genome. A key point in the method is the two-step preparation of the gel used for PFGE, where we include a low melting point agarose that enables further recovery of long DNA molecules from the gel. The study presents detailed NGS data of a global PFGE separation map of NotI digested human genomic DNA from IMR-90 cells. As we observed that some genomic regions were not separated according to the theoretical prediction, NGS can help to specify where the region of interest is located on a PFGE lane.

Single-molecule DNA optical mapping that enables assembly of genomes and detection of SVs was presented previously by other groups^6,8,40–43^. For example, Teague *et al*.^43^ described the analysis of SVs in four human genomes by using shotgun single-molecule restriction optical mapping and presented an impressive number of 4,205 of previously unreported SVs. However, as their analysis was based on 10 kb long fragments, the assembly of many single-molecule maps at each locus of a consensus map was required. In our method, multifold coverage of the genome and construction of consensus maps are not required because the probability of aligning a DR map from a single 100 kbp molecule to an incorrect position in the human reference genome is negligible^11^.

The new design of our previous nanofluidic device allowed capturing of DNA molecules as short as 0.7 Mb, which significantly increased the number of imaged molecules compared with previously published reports^11,28^. This number can be further improved, as automation of single DNA molecules capturing and visualization in a nanofluidic device has been demonstrated^44^. The throughput of the flow-stretch device is still limited compared to the commercially available Irys System (BioNano Genomics), which is based on stretching the DNA through confinement in nanochannels, and allows imaging of several gigabases of DNA per hour, but at a somewhat lower stretching. The enrichment method presented here could, however, potentially be improved by applying various restriction enzymes or other genomic editing tools such as the CRISPR/Cas9 system^45^. Moreover, we have demonstrated recently that cheap, disposable polymer devices (3 USD per device) can be used for single-molecule optical mapping. The high-yield production potential allowing several thousands of chips to be produced within a few days^28,46^ will greatly facilitate future optical mapping studies of megabase-size DNA molecules. Additionally, in contrast to many other optical mapping methods, there is a possibility to recover DNA molecules for further analysis^11^. The enrichment method presented here can also be used for sample preparation for commercial optical mapping platforms. Limiting the input DNA to specific genomic regions decreases the time and effort needed for mapping and data analysis.

One application for the enrichment method is exemplified by molecule M1 (Fig. 7), where we can identify molecules that flank and reach into the unannotated part of the genome, and thus allow the visualization of these regions that are extremely difficult to analyse with other DNA sequencing methods. Another possible application of the enrichment method could be the investigation of structural variations in large gene regulatory regions. Approximately 25% of the human genome contains gene-poor regions larger than 500 kb, named gene deserts^47^, many of which are evolutionarily conserved since they contain arrays of long-range *cis*-regulatory sequences that regulate key developmental genes which can be located more than 1 Mb away^47–51^. The disruption of such *cis*-regulatory domains by structural chromosomal rearrangements have been associated with long-range position effects in a number of human disorders and mouse models^52,53^. 112 out of 168 evolutionarily stable gene deserts^50^ overlap more than 80% with NotI fragments longer than 1.5 Mb. This is exemplified by a stable gene desert on chromosome 12 covered by four DR-maps from the combined fractions 4 and 5, and by NGS reads from fraction 4 (Supplementary Fig. S4). This domain regulates the developmental gene *LMO3* which has been reported to be associated with the development and aggressiveness of neuroblastoma^54^. Investigation of such gene deserts is a well-suited application for the enrichment method as it requires long, intact DNA molecules.

## Methods

### Preparation of megabase-sized DNA fragments

Preparation of cell nuclei from IMR-90 female human fetal lung fibroblasts (ATCC^®^ CCL-186™) in agarose plugs was based on a previously published method^55^ with some modifications described in the Supplementary Information.

### Pulsed-field gel electrophoresis separation

The two-step gel preparation was developed for pulsed-field gel electrophoresis (Supplementary Information). The DNA in plugs was digested with NotI enzyme (NEB) for 4 hours at 37 °C (50 units of enzyme per plug). Pulsed-field gel electrophoresis was performed on 1% UltraPure™ Low Melting Point Agarose (Invitrogen) at 11 °C for 120 hours in 0.5xTBE buffer with following parameters: voltage, 50 V linear to 45 V; angle, from 110° linear to 100°; interval, from 5000 sec logarithmic to 1000 sec. The gel was stained with SYBR® Safe DNA Gel Stain (Invitrogen) and visualized using a Syngene transilluminator. PFGE lanes were sliced into 2 mm- or 4 mm wide agarose fractions for NGS or single-molecule denaturation mapping, respectively.

### Ion proton sequencing

Corresponding fractions from three neighbouring lanes, i.e., lanes with the same size distribution of DNA, were combined to get a sufficient amount of DNA for sequencing. DNA libraries were prepared with standard Ion Xpress™ Plus gDNA Fragment Library Preparation reagents and protocols (Life Technologies) and sequenced on the Ion Proton™ semiconductor sequencer (Life Technologies). Each chip was run with six samples. Reads from the Ion Proton sequencing were trimmed for adapter-sequences using cutadapt^56^ and aligned to the human reference genome hg19 with bwa^57^. Alignments were generated in BAM format and uploaded to the SeqMonk software (http://www.bioinformatics.bbsrc.ac.uk/projects/seqmonk/). The DataStore summary report from SeqMonk provides information about the analysed data stores (Supplementary Table S1). Probes (regions of interest) were defined as 50 kb non-overlapping regions covering the entire human reference genome.

### Optical mapping in a nanofluidic device

For optical mapping, the DNA in 4 mm wide agarose fractions was stained with YOYO-1 (Invitrogen) and partially denaturated to produce the DR maps (Supplementary Information). Basic design and working principle of the device used in the experiment is described elsewhere^11,28^. Compared to the previous device design^11,28^, the present device has been modified so that the slit length is shorter (200 µm) and the cross-shaped slit is narrower at its centre (see Supplementary Fig. S1). Thus shorter molecules (>200 µm) can be stretched and imaged. Stretched DNA molecules were imaged with an Olympus IX81-ZDC2 microscope equipped with 100x/1.40 oil UPlanSApo objective. Images were recorded with an Andor’s iXon3 897 camera using 50-ms exposure and maximum electron multiplying (EM) gain. Molecule images presented in this paper were prepared with ImageJ^58^ and stitched with the ImageJ plugin Stitching^59^. DR maps were aligned with the theoretical prediction for the melting profile of the hg19 human reference genome as previously described in^11,27,28^.

### Methylation data for IMR-90 cells

Single-base resolution DNA methylation data for IMR90 was downloaded from the UCSC Genome Browser (track name: Human_IMR90_Meth) by use of the UCSC Table Browser data retrieval tool^60^. Theoretical prediction of NotI digestion was performed *in silico* by use of Matlab scripts developed in-house.

## Electronic supplementary material

Supplementary Information
