# Supplementary material for: Enrichment of megabase-sized DNA molecules for single-molecule optical mapping and next-generation sequencing
Source: Sci Rep. 2017 Dec 20;7:17893. doi: 10.1038/s41598-017-18091-6 (PMC5738345; doi:10.1038/s41598-017-18091-6)
Supplement: Supplementary file 1 — Supplementary Information [file 41598_2017_18091_MOESM1_ESM.doc]

**Supplementary Information for**

**“Enrichment of megabase-sized DNA molecules for single-molecule optical mapping and next-generation sequencing”.**

Joanna M. Łopacińska-Jørgensen^1^, Jonas N. Pedersen^2^, Mads Bak^1^, Mana M. Mehrjouy^1^,
Kristian T. Sørensen^2^, Peter F. Østergaard^2^, Brian Bilenberg^3^, Anders Kristensen^2^,
Rafael J. Taboryski^2^, Henrik Flyvbjerg^2^, Rodolphe Marie^2^, Niels Tommerup^1^ and Asli Silahtaroglu^1,*^

^1^Department of Cellular and Molecular Medicine, Faculty of Health and Medical Sciences, University of Copenhagen, Blegdamsvej 3B, Copenhagen, 2200, Denmark

^2^Department of Micro- and Nanotechnology, Technical University of Denmark, Ørsteds Plads 345a, Kongens Lyngby, 2800, Denmark

^3^NIL Technology ApS, Diplomvej 381, Kongens Lyngby, 2800, Denmark

* Correspondence and requests for materials should be addressed to A.S. (e-mail: asli@sund.ku.dk).

**DENATURATION-RENATURATION MAPPING**

As initially demonstrated by Reisner at al, DNA denaturation mapping was based on obtaining a DNA barcode after imaging the YOYO-1 stained genomic DNA following partial denaturation^1^. In DR mapping, the DNA is renatured before imaging of the barcode. DR mapping relies on the thermodynamical difference between adenosine-thymine (AT) rich and guanine-cytosine (GC) rich DNA, where AT-rich regions melt at a lower temperature (*T_m_,*_AC_) than GC-rich regions (*T_m_,*_GC_) due to the different number of hydrogen bonds. DNA molecules are stained with the intercalating dye YOYO-1 (see main text Fig. 1), and heated to a temperature *T* between the melting temperature of AT- and GC-rich regions, *T_m_,*_AT_ < *T* <*T_m_,*_GC_, so the AT-rich regions melts and dissolves the dye, whereas the dye remains in the stable GC-rich regions. After cooling, a denaturation pattern of bright (GC-rich) and dark (AT-rich) regions remain fixed in the renaturated double-stranded DNA^2^. Single DNA molecules with DR patterns are loaded into the nanofluidic chip, stretched up to 98% of their contour length by a cross-flow, and imaged (main text Fig. 1). The nanofluidic chip used in the experiments consists of four arms arranged in a cross-geometry (main text Fig. 1F, and also Supplementary Fig. S1). Fluid enters the center of the cross from the upper and lower arms, and leaves through the left and right arms (main text Fig. 1G). By proper manipulation of the flows, it is possible to align and stretch a megabase-long DNA molecule to almost its full contour length. Figure 1H shows an example of a flow-stretched DNA molecule approximately 0.7 megabase long. Notice the black and white DR pattern on the DNA that resembles a barcode. It is the intensity profile of the barcode, which is used to locate the molecule within a reference genome^1–3^.

The software package Bubblyhelix (www.bubblyhelix.org)^4^ is used for calculation of the theoretical melting profile of any DNA sequence. From a comparison between the DNA’s intensity profile and the theoretical melting profile of a reference genome, it is possible to localize the origin of a specific molecule unambiguously within the human reference genome.

## Preparation of megabase-long, size-selected molecules

It was previously reported that pipetting, vortexing, rocking, freeze-thawing, ultrasonication and ultrafiltration might lead to physical fragmentation of the DNA^5^. Thus, we carried out most of the experimental steps with DNA embedded in plugs of low-melting agarose in order to minimize the breakage. In this way, we managed to obtain a high-concentration of megabase-long molecules, even after the harsh steps in the protocol including long exposure of the molecules to electric field during electrophoresis, treatment with *β-agarase* for melting the agarose plug, and transfer of DNA twice in solution. We found, however, that it is crucial to transfer the DNA in solution with wide-bore pipette tips and not to transfer the DNA to an empty tube but into the buffer. We also observed that it is important to work with a DNA concentration of 10-50 ng/µL, as DNA concentrations outside this range led to inconsistent YOYO labelling, and, consequently, low-quality DR patterns on the DNA.

IMR-90 female human fetal lung fibroblasts (ATCC® CCL-186™) were grown until confluence in Dulbecco’s modified Eagle’s medium with Glutamax (DMEM; GIBCO Life Technologies), 10% fetal bovine serum, and 1% penicillin-streptomycin (P/S; GIBCO Life Technologies). Cells were washed with 1xPBS, dissociated with trypsin/EDTA and centrifuged at 1100 rpm for 3 min. The supernatant was discarded and cells were resuspended in freshly prepared ice-cold nuclei isolation buffer (10 mM Trizma base, 80 mM KCl, 10 mM EDTA, 1 mM spermidine trihydrochloride, 1 mM spermine tetrahydrochloride, 0.5% Triton X-100, 0.15% (vol/vol) 2-mercaptoethanol). Cells were incubated on ice for 10 min with occasional gentle vibration and afterwards centrifuged at 2,200g, 4°C for 10 min. The nuclei pellet was resuspended an additional time in ice-cold nuclei isolation buffer. The supernatant was discarded and the nuclei pellet was resuspended in ice-cold homogenization buffer (HB, 10 mM Trizma base, 80 mM KCl, 10 mMEDTA, 1 mM spermidine trihydrochloride, 1 mM spermine tetrahydrochloride). The nuclei solution was mixed with an equal amount of 1.4% (wt/vol) UltraPure™ Low Melting Point Agarose (Invitrogen) in HB solution. The nuclei-agarose solution was pipetted into agarose plug molds, 100 μl per well (Bio-Rad) (3x10^5^ cells per plug). The molds were placed at 4°C until the agarose has gelled. The plugs were incubated for 24 h at 50°C in the lysis buffer (0.5 M EDTA (pH 9.0 – 9.4), 1% (wt/vol) sodium lauryl sarcosine, 0.3 mg ml^-1^ proteinase K (NEB). Next, the plugs were washed once in 20 volumes of ice-cold 0.5xTBE and then three times in 20 volumes of ice-cold 0.5xTBE containing 0.1 mM PMSF on ice, each wash for 1 h. In order to remove PMSF, the plugs were washed three times in 20 volumes of ice-cold 0.5xTBE. The plugs can be stored at 4°C for up to 3 months without substantial DNA degradation.

## Pulsed-field gel electrophoresis separation

*Two-step gel preparation.* As the goal of our enrichment method is to isolate and image megabase-long DNA molecules, a crucial step is to recover the molecules from the PFGE gel without breakage. One possibility to recover DNA from PFGE gel is to apply β-Agarase I enzyme to digest agarose. Digestion of agarose releases the trapped DNA and yields carbohydrate molecules, so the gel can no longer solidify. The optimal temperature for β-Agarase I is 42°C, and the enzyme can be heat inactivated at 65°C.

In general, a special type of agarose with high gel strength, high electrophoretic mobility, and high exclusion limit is used for pulsed-field gel electrophoresis. It gives faster separation and better resolution of high molecular weight DNA by PFGE compared to routinely used agarose. However, the melting point of such an agarose is usually between 85-90°C, which is not compatible with the use of β-Agarase I. Only low-melting point agarose could be used for β-Agarase I digestion as the solution must be liquid at the incubation temperature of 42°C. Gels prepared only from low-melting agarose (1% UltraPure™ Low Melting Point Agarose, Invitrogen) had a tendency to detach from the gel tray during electrophoresis and thereby terminate the experiment. Therefore, we developed a two-step gel preparation. In the first step, a 1% gel of 7 mm thickness was prepared by dissolving 3.0 g high melting point agarose (Certified™ Megabase Agarose, 1613108, BioRad) in 300 ml of 0.5xTBE buffer. After the gel solidification, a high melting point agarose frame is created by removing a 16 x 16 cm gel part. In the second step, 1% UltraPure™ Low Melting Point Agarose (Invitrogen) in 0.5xTBE buffer is poured into this frame on the gel tray (Supplementary Fig. S5).

DNA separation was compared in high melting and low melting agarose areas on the double-gel and a difference was observed in the distance DNA had migrated. (Supplementary Fig. S6). As size markers we used CHEF S. pombe (range 3.5 – 5.7 Mb, Sp) and CHEF DNA H. wingei (range 1.05 – 3.13 Mb, Hw) from Bio-Rad. In PFGE lanes for NotI digested human DNA, we observed “high-density” bands both in high melting and low melting point agarose (bands indicated with a red arrow on Supplementary Fig. S6). Similar bands were also observed in controls, i.e., plugs that were treated with NotI digestion solution without the enzyme. A possible explanation is that some DNA is damaged during the plug preparation.

*Preliminary pulsed-field gel electrophoresis.* In order to remove any damaged DNA, a preliminary pulsed-field gel electrophoresis was performed before restriction enzyme digestion. CHEF DNA H. wingei (range 1.05 – 3.13 Mb) and CHEF S. pombe (range 3.5 – 5.7 Mb) from Bio-Rad were used as size markers. Electrophoresis was performed at 13°C for 48 hours in 0.5xTBE buffer, using a Rotaphor apparatus (Biometra). Running parameters were set as follows: voltage, 190V linear to 175V; angle, from 130° linear to 115°; interval, from 150 sec logarithmic to 30 sec. The plugs were cut from the gel and washed once with ice-cold 0.5xTBE.

In order to digest DNA with restriction enzyme, the digestion mixture was prepared in a volume 200 µl per plug –, 50 U of NotI restriction enzyme (R0189, NEB), 20 µl of appropriate 10x restriction buffer, BSA and sterile water to make up the volume. The plugs were gently shaken at the recommended temperature for 4 hours. To stop the reaction, the digestion mixture was removed and plugs were washed in 20 volumes of ice-cold 0.5xTBE. The digestion of the DNA was performed shortly before running the second pulsed-field gel electrophoresis for the main separation.

*Second pulsed-field gel electrophoresis.* The second pulsed-field gel electrophoresis was then performed on 1% UltraPure™ Low Melting Point Agarose (Invitrogen) at 11°C for 120 hours in 0.5xTBE buffer, using a Rotaphor apparatus (Biometra). Running parameters were set as follows: voltage, 50V linear to 45V; angle, from 110° linear to 100°; interval, from 5000 sec logarithimic to 1000 sec. The gel was stained with SYBR® Safe DNA Gel Stain (Invitrogen) and visualized using a Syngene transilluminator (Supplementary Fig. S8).

PFGE lanes were sliced into 2 mm or 4 mm wide agarose fractions for NGS or single-molecule denaturation mapping, respectively. For the NGS analysis, 11 fractions were defined: plug and fractions 1-10. For optical mapping, the DNA from combined fractions 4 and 5 was the main source of single molecules from IMR-90 cells.

Supplementary Figure S8a shows a plot of the intensity profile along the arrows marked in main text Figure 2a. We use DNA ladders from *S. pombe* and *H. wingei* to calibrate the fragment size as function of the position in the gel. As the number of DNA ladders and their lengths are known, we fit the same number of Gaussian distributions to the two intensity profiles (3 for *S. Pombe* and 4 for *H. wingei*, Supplementary Fig. S8a). Supplementary Figure S8b shows the centers of the fitted Gaussian profiles versus the lengths of the ladders.

## Optical mapping in a nanofluidic device

For optical mapping, the DNA in 4 mm wide agarose fractions was stained with YOYO-1 (Invitrogen) with a basepair: dye ratio of 5:1 according to the Invitrogen’s protocol. The solution was incubated for 1 h at room temperature, followed by 1 h at 50°C. The agarose pieces were washed once with 0.5xTBE, the buffer was removed, and the pieces were melted at 67°C for 10 min. The solution was cooled to 42°C over a period of 2 min and 4 μl of β-agarase (1000 U ml^−1^, New England Biolabs, MA, USA) was added. The samples were incubated at 42°C for 3 h and stored at 4°C. Then the YOYO-1 stained DNA molecules were partially denaturated by using a 2720 Thermal Cycler (Applied Biosystems) to produce the DR maps (see main text Fig. 1), and then transferred from the tube to the inlet of the nanofluidic device.

## Repeated denaturation-renaturation patterns

Another application of DR maps is for detection of repeated elements on the kb-scale. Many of the 40 DR maps that could not be aligned to the reference genome contained regions with clearly visible repeating blocks or regions. As an example a sequence with 25 repeated elements is shown (Supplementary Fig. S8). A Fourier decomposition of the intensity gives a repeat length of 2 µm. In the chip, the DNA is stretched to approximately 90% of its contour length^2^, so this repeat length corresponds approximately to 6.5 kb as the length of a basepair is 0.34 nm. Such repeats cannot be resolved with NGS sequencing due their short read lengths and the alignment difficulties (<300 bp)^6^, but the repeats are obvious in the DR maps.

## References

1. Reisner, W. *et al.* Single-molecule denaturation mapping of DNA in nanofluidic channels. *Proc. Natl. Acad. Sci.* **107,** 13294–13299 (2010).

2. Marie, R. *et al.* Integrated view of genome structure and sequence of a single DNA molecule in a nanofluidic device. *Proc. Natl. Acad. Sci. U. S. A.* **110,** 4893–8 (2013).

3. Østergaard, P. F. *et al.* Optical mapping of single-molecule human DNA in disposable, mass-produced all-polymer devices. *J. Micromechanics Microengineering* **25,** 105002 (2015).

4. Tøstesen, E., Liu, F., Jenssen, T.-K. & Hovig, E. Speed-up of DNA melting algorithm with complete nearest neighbor properties. *Biopolymers* **70,** 364–376 (2003).

5. Yoo, H.-B. Flow cytometric investigation on degradation of macro-DNA by common laboratory manipulations. *J. Biophys. Chem.* **2,** 102–111 (2011).

6. Norris, A. L., Workman, R. E., Fan, Y., Eshleman, J. R. & Timp, W. Nanopore sequencing detects structural variants in cancer. *Cancer Biol. Ther.* **17,** 246–253 (2016).

**Supplementary Figure S1. Schematics of the flow-stretch device and formation of the DR map.** *(A)* The device has four microchannels connected through a cross-shaped nanoslit. The DNA is loaded in one of the inlets. *(B)* The cross-shaped nanoslit creates a double-elongation flow when pressure is applied to the vertical arms. DNA is elongated in the horizontal arms. *(C)* DNA is imaged in four overlapping field-of-views (FOV). The fluorescence barcode is extracted from each FOV image. Then the four barcodes are stitched together to a final DR map.

**Supplementary Figure S2. Cumulative size distribution of the NotI fragments defined in the main text.** In total, 13.5% of the NotI fragments are longer than 1 Mb.

**Supplementary Figure S3.** **Helicity (fraction of closed basepairs) versus temperature for the centromeric region (44.7-45.7 Mb) and the two regions flanking it on chromosome 8.** For the centromeric region, the helicity drops abruptly between 73C and 74C. So the DNA melts completely at temperatures above 74C. In contrast, the neighbouring regions show a gradual decrease of the helicity with temperature.

**Supplementary Figure S4. GenomeBrowser visualization of the chr12:15,000,000-19,000,000 region.** The NotI fragment is displayed as a red bar, DR maps from the pooled fractions 4 and 5 are presented as blue bars. The NGS island from the fraction 4 is marked as a black bar, whereas the stable gene desert is shown as a purple bar.

***
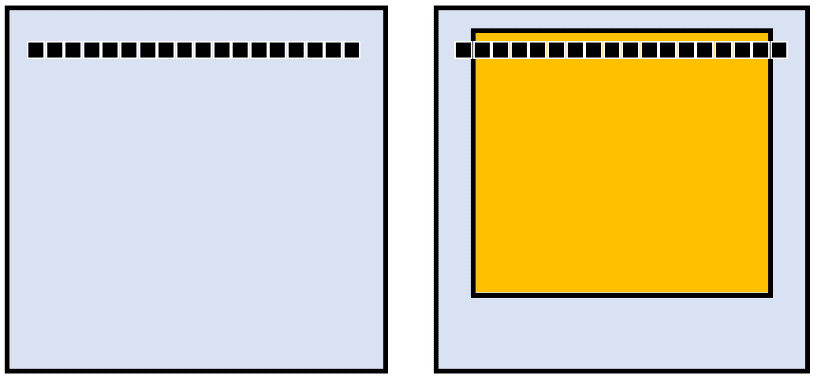
***

**Supplementary Figure S5. Double-step gel preparation for PFGE separation of NotI digested human DNA.** (Left) High melting point agarose gel is prepared by dissolving 3.0 g agarose (Certified™ Megabase Agarose, 1613108, BioRad) in 300 ml of 0.5xTBE buffer. The size of gel tray is 20x20 (cm)^2^. (Right) 16x16 cm gel part (yellow) is removed and a high melting point agarose frame is created. Next, 1% UltraPure™ Low Melting Point Agarose (Invitrogen) in 0.5xTBE buffer is poured into this frame on the gel tray.

**Supplementary Figure S6*.* Comparing DNA separation.** High melting point agarose (left) and low melting agarose gels (right).


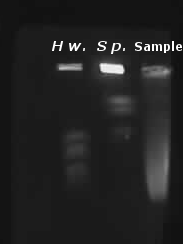


**Supplementary Figure S7. Unprocessed gel image of the separation of NotI-cut DNA related to Figure 2.**

**Supplementary Figure S8. NotI-cut DNA extracted from gel.** (a) Intensity profile along the arrows in main text Figure 2a. Gaussians corresponding to the number of ladders are fitted to the intensity profile. (b) Distance travelled in the gel versus the length of ladders obtained from the centers of the fitted Gaussian distributions. Inset: same data on a logarithmic scale. Full line is a fit to an exponential function.

**Supplementary Figure S9. Example of a molecule with repeated elements.** Upper panel shows a microscopy image of the molecule (scale bar is 10 um), and the lower panels shows the intensity profile along the molecule. The period of the repeated pattern is approximately 2 µm, i.e.~6.5 kb.

**Supplementary Table S1.** Number of molecules overlapping with the same position in the reference genome.

| Coverage | 1x coverage | 2x coverage | 3x coverage | 4x coverage |
| --- | --- | --- | --- | --- |
| Number of regions | 505 | 151 | 34 | 5 |

**Supplementary Table S2.** DataStore summary report from SeqMonk.

| **Data Store** | **Total Read Count** | **Forward Read Count** | **Reverse Read Count** | **Mean Read Length** | **Total Read Length** |
| --- | --- | --- | --- | --- | --- |
| **plug_hg19.bam** | 5,991,176 | 2,993,192 | 2,997,984 | 150 | 901,657,540 |
| **frac1_hg19.bam** | 8,607,021 | 4,302,629 | 4,304,392 | 152 | 1,309,255,113 |
| **frac2_hg19.bam** | 5,161,846 | 2,581,558 | 2,580,288 | 153 | 793,167,908 |
| **frac3_hg19.bam** | 12,603,559 | 6,288,213 | 6,315,346 | 157 | 1,979,275,922 |
| **frac4_hg19.bam** | 10,214,399 | 5,100,591 | 5,113,808 | 148 | 1,517,340,960 |
| **frac5_hg19.bam** | 10,821,134 | 5,406,947 | 5,414,187 | 153 | 1,656,678,231 |
| **frac6_hg19.bam** | 11,225,656 | 5,614,556 | 5,611,100 | 145 | 1,635,482,464 |
| **frac7_hg19.bam** | 4,793,887 | 2,397,876 | 2,396,011 | 154 | 742,791,281 |
| **frac8_hg19.bam** | 6,377,422 | 3,187,797 | 3,189,625 | 160 | 1,020,478,324 |
| **frac9_hg19.bam** | 6,581,192 | 3,289,821 | 3,291,371 | 154 | 1,019,022,155 |
| **frac10_hg19.bam** | 6,174,317 | 3,087,469 | 3,086,848 | 152 | 939,933,376 |
